# Elevated Prorenin Induces Podocyte Injury and Glomerular Fibrosis in cyp1a1-Prorenin Transgenic Rats

**DOI:** 10.3390/ijms27177888

**Published:** 2026-09-03

**Authors:** Chunyan Gu, Xia Liu, Jie Wu, Yufeng Huang

**Affiliations:** 1 Division of Nephrology & Hypertension, Department of Internal Medicine, University of Utah Health, Salt Lake City, UT 84108, USA; guchunyan@njucm.edu.cn (C.G.); liuxia_fd@ntu.edu.cn (X.L.); wujie@cpu.edu.cn (J.W.); 2 Department of Pathophysiology, College of Medicine, University of Nantong, Nantong 226001, China

**Keywords:** prorenin, podocyte injury, glomerular fibrosis, diabetic nephropathy, chronic kidney disease

## Abstract

Plasma prorenin is commonly elevated in patients with diabetes and has been associated with the development of albuminuria and progression of diabetic nephropathy. Because albuminuria often reflects podocyte injury, the pathogenic role of prorenin in podocyte dysfunction warrants further investigation. In this study, we examined the association between prorenin and podocyte injury, as well as glomerular fibrosis, using a transgenic rat model in which prorenin is inducibly expressed and secreted from the liver. cyp1a1-prorenin transgenic rats were randomized to receive diets containing increasing concentrations of the gene activator indole-3-carbinol (I3C; 0.05%, 0.15%, or 0.3%) for 4 weeks. Wild-type rats maintained on a normal diet served as controls. I3C administration resulted in a dose-dependent increase in plasma prorenin levels in transgenic rats. Elevated prorenin was associated with increased mean arterial pressure and urinary albumin excretion, accompanied by a dose-dependent reduction in podocyte number and slit diaphragm protein expression, as well as segmental foot process effacement and podocyte hypertrophy. In addition, increased prorenin stimulated renal expression of profibrotic factors and promoted glomerular fibrosis. Treatment with either amlodipine or enalapril for 6 weeks prevented the development of hypertension and partially attenuated podocyte injury, albuminuria, and renal fibrosis, without fully reversing these changes. These protective effects were associated with suppression of NF-κB- and Nox2-mediated inflammatory and oxidative stress pathways. Collectively, these findings demonstrate that prorenin promotes podocyte injury and glomerular fibrosis through mechanisms that are partially dependent on hypertension and angiotensin II but also involve angiotensin II-independent pathways.

## 1. Introduction

Elevated plasma prorenin is commonly found in patients with diabetes and has long been associated with microvascular complications, including diabetic nephropathy (DN) [1,2,3,4,5]. Notably, analysis of the Chronic Renal Insufficiency Cohort (CRIC) study demonstrated that higher plasma prorenin levels are independently associated with diabetes and reduced glomerular filtration rate (eGFR), further supporting the clinical relevance of prorenin in CKD progression [6]. Despite these associations, the mechanisms responsible for increased circulating prorenin in diabetes remain poorly understood, and the direct contribution of prorenin to the pathogenesis of DN has not been fully established.

The (pro)renin receptor (PRR, ATP6AP2) has been identified as a key mediator of prorenin signaling [7,8]. Binding of prorenin to PRR not only enhances local renin–angiotensin system activity but also activates angiotensin II-independent intracellular signaling pathways, including the PLZF–PI3K–p85α axis, MAPK signaling cascades, and TGF-β signaling [7,9,10]. These pathways promote oxidative stress, inflammatory responses, cytoskeletal disorganization, and apoptosis in multiple cell types. PRR is highly expressed in podocytes, where it plays an essential role in maintaining cellular homeostasis and survival [11,12]. In vitro studies have shown that podocyte-specific PRR deficiency or pharmacological inhibition of vacuolar H+-ATPase activity, which is closely associated with PRR function, disrupts intracellular vesicle acidification and leads to podocyte dysfunction and cell death [12]. In contrast, accumulating evidence indicates that pathological activation of the prorenin–PRR axis contributes to podocyte injury. In cultured podocytes and experimental models of diabetic nephropathy, inhibition of PRR using siRNA or a handle-region peptide (HRP) antagonist attenuates hyperglycemia-induced oxidative stress, inflammation, and podocyte injury, resulting in reduced albuminuria and glomerular damage [13,14,15]. These findings suggest that excessive prorenin–PRR signaling contributes to the development of diabetic kidney disease. However, it remains unclear whether the elevated circulating prorenin observed in diabetes directly induces podocyte injury and glomerular disease through PRR-dependent mechanisms in vivo.

To address this question, we generated transgenic rats with inducible liver-specific expression of rat prorenin under the control of the cytochrome P450 1A1 (cyp1a1) promoter [10]. Transgene expression was activated by indole-3-carbinol (I3C), a naturally occurring compound found in cruciferous vegetables that acts through the aryl hydrocarbon receptor and possesses a short biological half-life [16]. Using this model, we sought to determine whether chronic elevation of circulating prorenin is sufficient to induce podocyte injury and glomerular fibrosis in vivo, independent of hyperglycemia, and to define the contribution of PRR-mediated signaling to these pathological responses.

## 2. Results

Study 1: Dose-dependent effects of prorenin on podocyte injury.

### 2.1. Inducible Prorenin Expression

The inducible cyp1a1-prorenin transgenic rat model, in which the cytochrome P450 promoter cyp1a1 drives liver-specific expression of prorenin, has been established and characterized previously [10]. The cyp1a1 promoter is not constitutively active but can be strongly induced by xenobiotics such as indole-3-carbinol (I3C), a compound with a short biological half-life [16,17,18]. Effective induction of cyp1a1-driven transgene expression by dietary I3C has been validated in this model [10].

**Figure 1:**
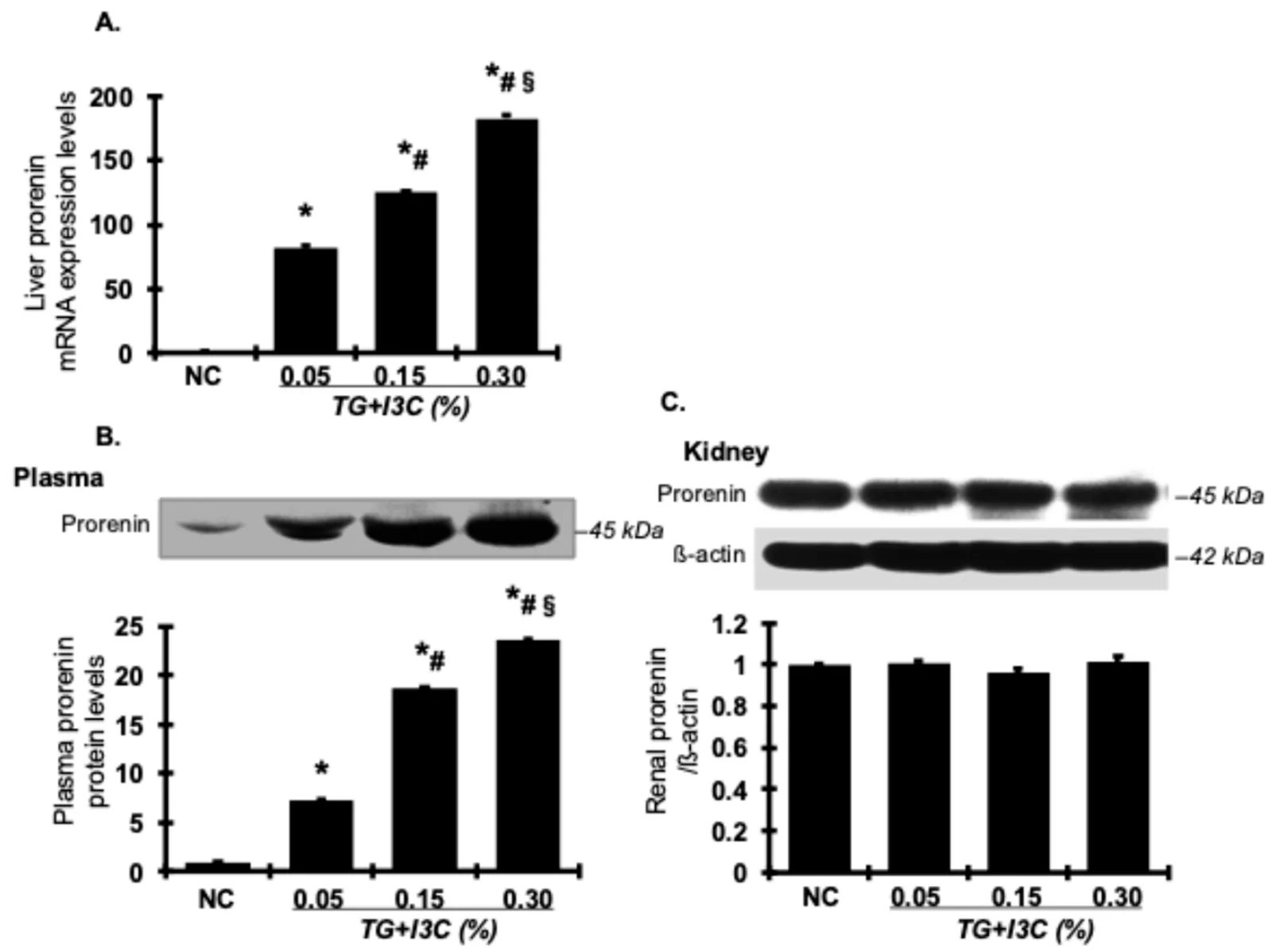
Dose-dependent induction of prorenin overexpression in the liver and circulation, but not the kidney, in cyp1a1-prorenin transgenic rats. (**A**) Cyp1a1-prorenin transgenic rats (TG) were administered increasing concentrations of indole-3-carbinol (I3C) to induce prorenin transgene expression in the liver tissue, which was quantified by real-time RT-PCR and normalized to β-actin expression. (**B**) Representative Western blot analyses elucidate a dose-dependent increase in prorenin protein expression in plasma of transgenic rats following I3C treatment. (**C**) Representative Western blot analyses of prorenin and β-actin protein expression in renal cortex tissue show no significant differences among groups. Quantitative analyses are presented in the corresponding panels. Data are presented as mean ± SD. * *p* < 0.05 vs. normal controls; # *p* < 0.05 vs. 0.05% I3C-induced transgenic (TG) rats; § *p* < 0.05 vs. 0.15% I3C-induced TG rats. n = 4–6 per group.

To examine the dose-dependent effects of prorenin induction, transgenic rats were fed diets containing increasing concentrations of I3C (0.05%, 0.15%, or 0.3%) for 4 weeks. I3C administration resulted in a dose-dependent increase in hepatic prorenin mRNA expression (Figure 1A). This was accompanied by a significant, dose-dependent elevation of plasma prorenin levels (Figure 1B), whereas prorenin expression in the kidney remained unchanged (Figure 1C), as determined by Western blot analysis. These findings confirm that I3C induction drives dose-dependent overexpression of prorenin in the liver, leading to increased circulating levels of unprocessed prorenin without local renal overexpression, consistent with our previous observations [10].

### 2.2. Dose-Dependent Effects of Elevated Prorenin on Clinical Parameters

As shown in Table 1, transgenic (TG) rats fed diets containing 0.05% and 0.15% I3C exhibited reduced body weight gain over 4 weeks compared with wild-type (WT) controls. Notably, TG rats receiving the highest dose of I3C (0.3%) exhibited significant weight loss (~58.43 g), whereas WT rats showed a gain of ~89.06 g over the same period.

At baseline, systolic blood pressure (SBP), measured by tail-cuff plethysmography, did not differ among groups, and non-induced cyp1a1-prorenin TG rats remained normotensive. In contrast, I3C induction markedly increased mean arterial pressure (MAP), rising from 80.6 to 137.8 mmHg in TG rats (Table 1), indicating that elevated circulating prorenin induces hypertension.

Plasma blood urea nitrogen (BUN) levels in induced TG rats were significantly elevated in I3C-induced TG rats, compared with normal controls, indicating impaired renal function (Table 1). Creatinine clearance (Ccr) analysis showed a biphasic trend: low-dose I3C induction was associated with increased Ccr level, suggestive of hyperfiltration, whereas higher I3C doses (≥0.15%) were associated with a decline in Ccr level. However, these changes did not reach statistical significance, likely due to limited sample size and variability in measurements.

At the end of the 4-week induction period, urinary albumin-to-creatinine (A/C) ratio was markedly elevated (>10-fold) in I3C-induced TG rats compared with TG rats prior to prorenin induction. In contrast, WT controls showed no change in A/C ratio following I3C administration. These findings indicate that elevated circulating prorenin leads to hypertension, renal dysfunction, and albuminuria, although the magnitude of these effects was not strictly dose-dependent.

Furthermore, I3C induction resulted in a dose-dependent increase in kidney weight/body weight and heart weight/body weight ratios in TG rats compared with WT controls, consistent with renal and cardiac hypertrophy.

### 2.3. Dose-Dependent Effects of Elevated Prorenin on Podocyte Injury

**Figure 2:**
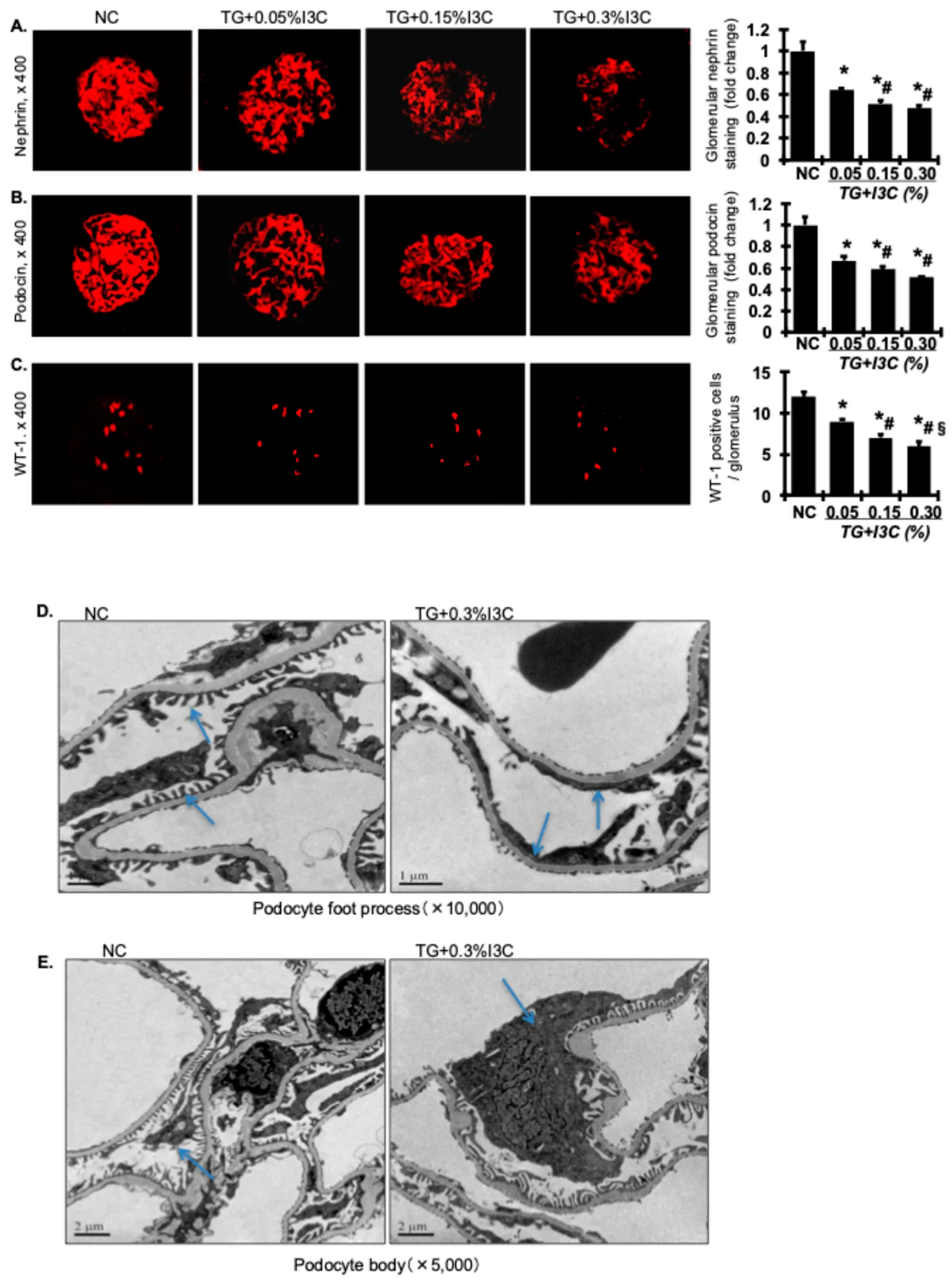
Elevated circulating prorenin induces dose-dependent podocyte injury in cyp1a1-prorenin transgenic rats. (**A**–**C**) Representative immunofluorescence staining of nephrin ((**A**), top panels), podocin ((**B**), middle panels), and WT-1-positive podocyte nuclei ((**C**), bottom panels) in glomeruli from wild-type control rats and cyp1a1-prorenin transgenic rats treated with increasing concentrations of indole-3-carbinol (I3C) to induce prorenin overexpression. Quantitative analyses of nephrin and podocin fluorescence intensity and WT-1-positive cell counts are shown in the corresponding panels. Data are presented as mean ± SD. * *p* < 0.05 vs. normal controls; # *p* < 0.05 vs. 0.05% I3C-induced transgenic (TG) rats; § *p* < 0.05 vs. 0.15% I3C-induced TG rats. (**D**,**E**) Representative transmission electron microscopy (TEM) images further revealed that transgenic rats exhibited segmental foot process effacement, widening of foot processes (**D**) (as arrows pointed), and podocyte hypertrophy (**E**) (as arrows pointed), compared with normal control animals. n = 4–6 per group.

Immunofluorescence (IF) staining demonstrated that 4 weeks of I3C induction resulted in a dose-dependent reduction in slit diaphragm proteins, including nephrin and podocin, as well as a decrease in the number of podocytes (WT-1–positive cells) per glomerular area in cyp1a1-prorenin transgenic (TG) rats compared with wild-type (WT) controls (Figure 2A–C). These changes are consistent with the development of albuminuria observed in I3C-induced TG rats. Transmission electron microscopy (TEM) analysis of TG rats receiving the highest dose of I3C (0.3%) revealed markedly segmental podocyte foot process effacement and podocyte hypertrophy compared with WT controls (Figure 2D,E).

**Figure 3:**
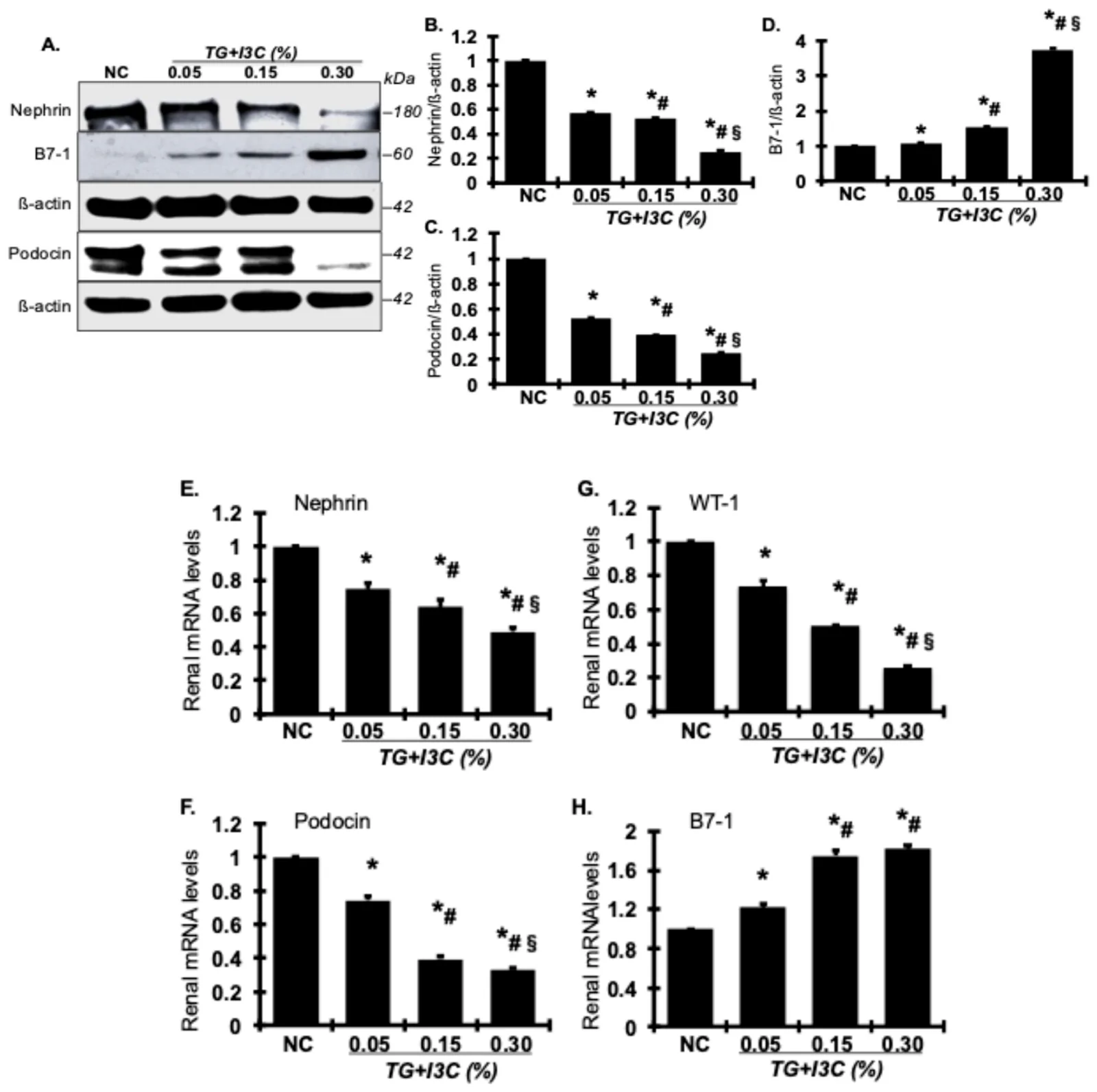
Elevated circulating prorenin alters the expression of podocyte injury-markers in cyp1a1-prorenin transgenic rats. (**A**) Representative Western blot analyses of podocyte injury-related proteins, nephrin, podocin, and B7-1, in kidney cortex lysates from wild-type control rats and cyp1a1-prorenin transgenic rats treated with increasing concentrations of indole-3-carbinol (I3C) to induce prorenin overexpression. β-actin was used as a loading control. (**B**–**D**) Quantitative densitometric analyses of the corresponding Western blots shown in panel A. (**E**–**H**) Relative renal mRNA expression of nephrin, podocin, WT-1, and B7-1 in wild-type and prorenin transgenic rats, determined by real-time RT-PCR and normalized to β-actin expression. Data are presented as mean ± SD. * *p* < 0.05 vs. normal controls; # *p* < 0.05 vs. 0.05% I3C-induced transgenic (TG) rats; § *p* < 0.05 vs. 0.15% I3C-induced TG rats. n = 4–6 per group.

Consistent with these findings, Western blot analysis showed a dose-dependent decrease in nephrin and podocin expression, along with a dose-dependent increase in the podocyte injury marker B7-1 in renal cortical tissue of I3C-induced TG rats (Figure 3A–D). Similarly, real-time RT-PCR analysis demonstrated reduced mRNA expression of nephrin, podocin, and WT-1, accompanied by increased B7-1 expression (Figure 3E–H).

Collectively, these results indicate that elevated circulating prorenin induces podocyte injury, contributing to the development of albuminuria.

### 2.4. Dose-Dependent Effects of Elevated Prorenin on Glomerular Fibrosis

**Figure 4:**
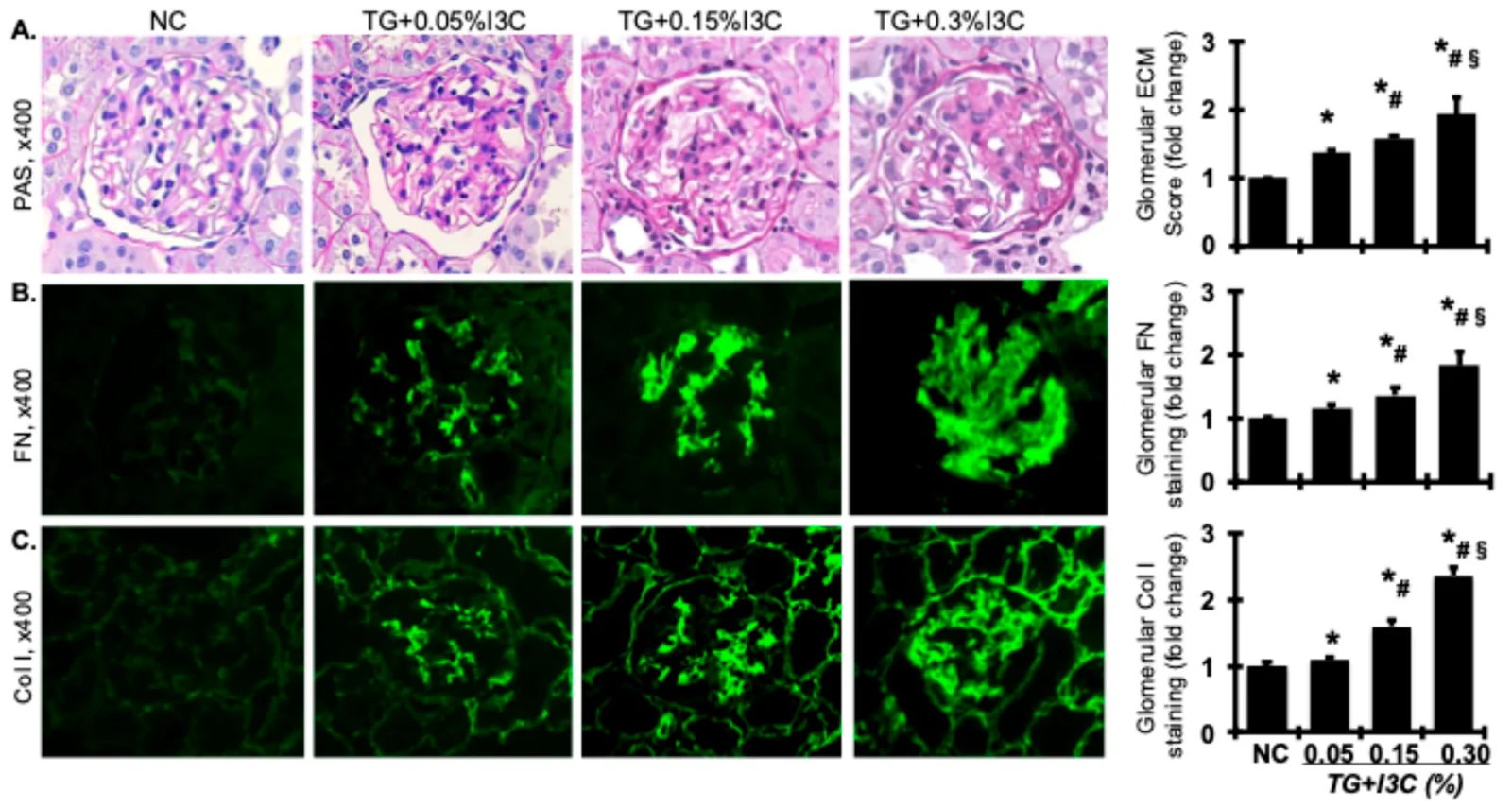
Elevated circulating prorenin induces dose-dependent glomerular fibrosis in cyp1a1-prorenin transgenic rats. (**A**–**C**) Representative photomicrographs of periodic acid–Schiff (PAS) staining (**A**), top panels), fibronectin (FN) immunofluorescence staining (**B**), middle panels), and collagen I (Col-I) immunofluorescence staining (**C**), bottom panels) in kidneys from wild-type control rats and cyp1a1-prorenin transgenic rats treated with increasing concentrations of indole-3-carbinol (I3C) to induce prorenin overexpression. Quantitative analyses of glomerular injury scores and fibrotic marker deposition are presented in the corresponding panels. Data are presented as mean ± SD. * *p* < 0.05 vs. normal controls; # *p* < 0.05 vs. 0.05% I3C-induced transgenic (TG) rats; § *p* < 0.05 vs. 0.15% I3C-induced TG rats. n = 4–6 per group.

As shown in Figure 4A–C, increased prorenin levels induced dose-dependent glomerular extracellular matrix accumulation, including fibronectin (FN) and collagen I (Col I) deposition, as demonstrated by PAS and immunofluorescence staining.

**Figure 5:**
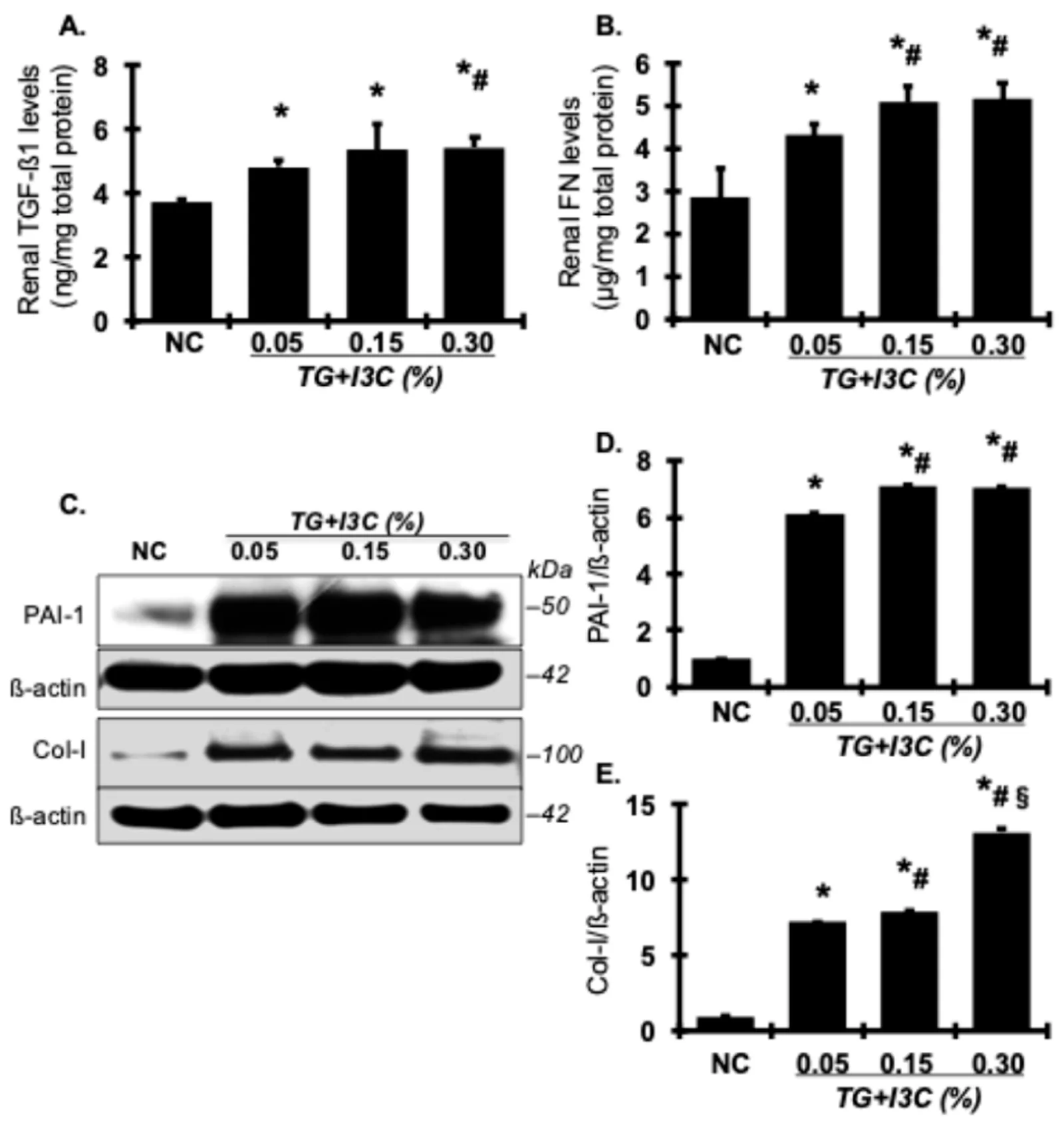

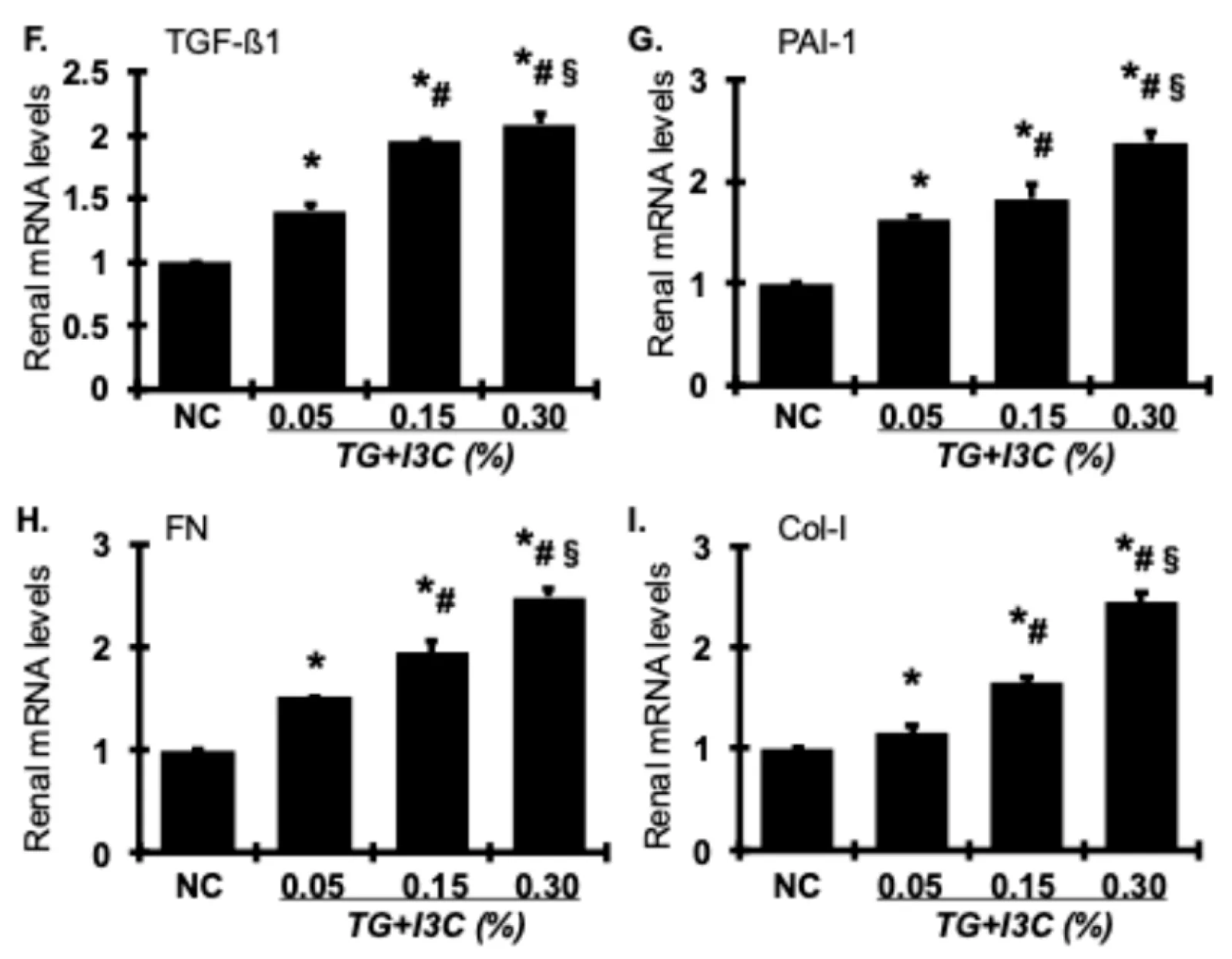
Elevated circulating prorenin induces dose-dependent increases in profibrotic marker expression in cyp1a1-prorenin transgenic rats. (**A**,**B**) Renal cortex levels of TGF-β1 and FN were determined by ELISA. (**C**) Representative Western blot analyses of PAI-1, Col-I, and β-actin protein expression in renal cortex tissues. β-actin was used as a loading control. (**D**,**E**) Quantitative densitometric analyses of the corresponding Western blots shown in panel (**C**). (**F**–**I**) mRNA expression levels of TGF-β1, PAI-1, FN and Col-I in renal cortex tissue were determined by real-time RT-PCR and normalized to β-actin expression. Data are presented as mean ± SD. * *p* < 0.05 vs. normal controls; # *p* < 0.05 vs. 0.05% I3C-induced transgenic (TG) rats; § *p* < 0.05 vs. 0.15% I3C-induced TG rats. n = 4–6 per group.

Further analysis of profibrotic mediators by ELISA and Western blot revealed that renal transforming growth factor-β1 (TGF-β1) (Figure 5A) and plasminogen activator inhibitor-1 (PAI-1) (Figure 5C,D), two key profibrotic factors, were significantly elevated even at the lowest dose of I3C induction, with progressive increases observed at intermediate and high doses. Similarly, fibrotic protein markers, including FN (Figure 5B) and Col I (Figure 5C,E), were significantly upregulated in response to prorenin overexpression. Among these, FN showed progressive increases with higher I3C doses and remained elevated at the highest dose, whereas Col I expression was most prominently increased at the highest I3C dose.

Consistent with these findings, mRNA expression levels of these fibrotic markers in renal tissue also increased in a dose-dependent manner compared with non-transgenic controls (Figure 5F–I). Together, these results demonstrate that elevated circulating prorenin promotes dose-dependent glomerular fibrosis.

Study 2: Effect of amlodipine (AM) and enalapril (Ena) on prorenin-induced podocyte injury and glomerular fibrosis.

### 2.5. Effect of Treatment on Plasma Prorenin Levels

Based on the results from Study 1, the intermediate dose of I3C (0.15%) was selected to induce prorenin overexpression in cyp1a1–prorenin transgenic rats. Using this model, we evaluated the effects of amlodipine (AM) and enalapril (Ena), administered at maximally effective doses [19,20,21] for 6 weeks, beginning at the same time as prorenin overexpression induction with I3C to distinguish hypertension-dependent and Ang II-dependent mechanisms underlying prorenin-induced podocyte injury and glomerular fibrosis.

**Figure 6:**
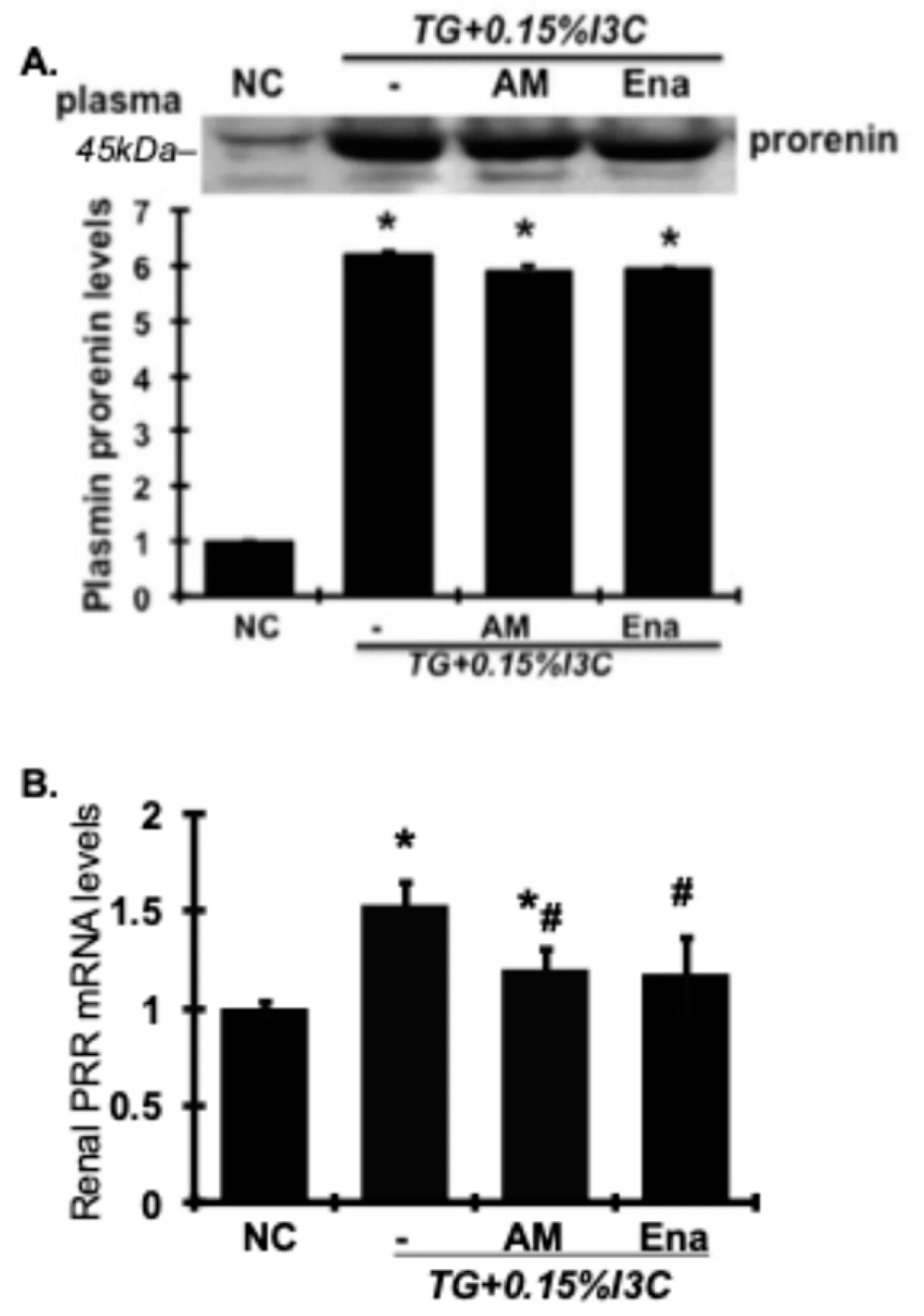
Effect of treatment with amlodipine and enalapril on circulating prorenin levels and renal prorenin receptor (PRR) expression in cyp1a1-prorenin transgenic rats. (**A**) Representative Western blot analyses of prorenin protein expression in plasma from normal control and prorenin-overexpressing rats (0.15% I3C-induced transgenic rats (TG)) treated with or without amlodipine or enalapril. Quantitative densitometric analyses of the corresponding Western blots are shown below. (**B**) mRNA expression levels of PRR in renal cortex tissue were determined by real-time RT-PCR and normalized to β-actin expression. Data are presented as mean ± SD. * *p* < 0.05 vs. normal control (NC); # *p* < 0.05 vs. untreated prorenin-transgenic rats (TG + 0.15% I3C). n = 5–6 per group.

Consistent with Study 1, induction with 0.15% I3C for 6 weeks resulted in a marked increase in plasma prorenin levels in transgenic rats, reaching approximately sixfold higher levels than those in wild-type controls. This elevation was not altered by treatment with either AM or Ena (Figure 6A). Interestingly, renal PRR mRNA expression was significantly increased (~50%) in prorenin-overexpressing rats compared with wild-type controls. Treatment with AM significantly attenuated this increase, whereas Ena nearly normalized PRR expression to control levels (Figure 6B).

### 2.6. Effect of Treatment on Clinical Characteristics

As shown in Table 2, prorenin-overexpressing rats exhibited significantly less body weight gain over the 6-week study period than wild-type (WT) controls, whereas WT rats gained an average of 134.44 g. Treatment with either AM or enalapril (Ena) attenuated this reduction in body weight gain. Baseline systolic blood pressure did not differ among the groups before induction of prorenin overexpression. However, prorenin overexpression markedly increased blood pressure, as reflected by elevated mean arterial pressure (MAP) over the 6-week period. This increase in MAP was effectively prevented by treatment with either AM or Ena.

Plasma BUN levels were elevated in prorenin-overexpressing rats, indicating impaired renal function, and were partially reduced by Ena treatment. Although AM treatment showed a trend toward decreased BUN levels, this did not reach statistical significance. Creatinine clearance (Ccr) exhibited a decreasing trend in prorenin transgenic rats and a recovery trend in treated groups; however, only Ena-treated rats showed a statistically significant increase compared with untreated prorenin-overexpressing rats, while overall variability remained high (Table 2). Compared with non-transgenic controls, prorenin-overexpressing rats developed significant albuminuria, as evidenced by increased 24 h urinary albumin excretion (UAE) and urine albumin-to-creatinine (A/C) ratio. These changes were significantly attenuated by both AM and Ena, with Ena showing greater efficacy than AM. Kidney and heart hypertrophy observed in prorenin transgenic rats were also reduced by both treatments (Table 2).

Notably, while AM and Ena prevented hypertension and partially improved albuminuria and renal dysfunction, they did not fully normalize these parameters or prevent cardiac and renal hypertrophy.

### 2.7. Effect of Treatment on Podocyte Injury

**Figure 7:**
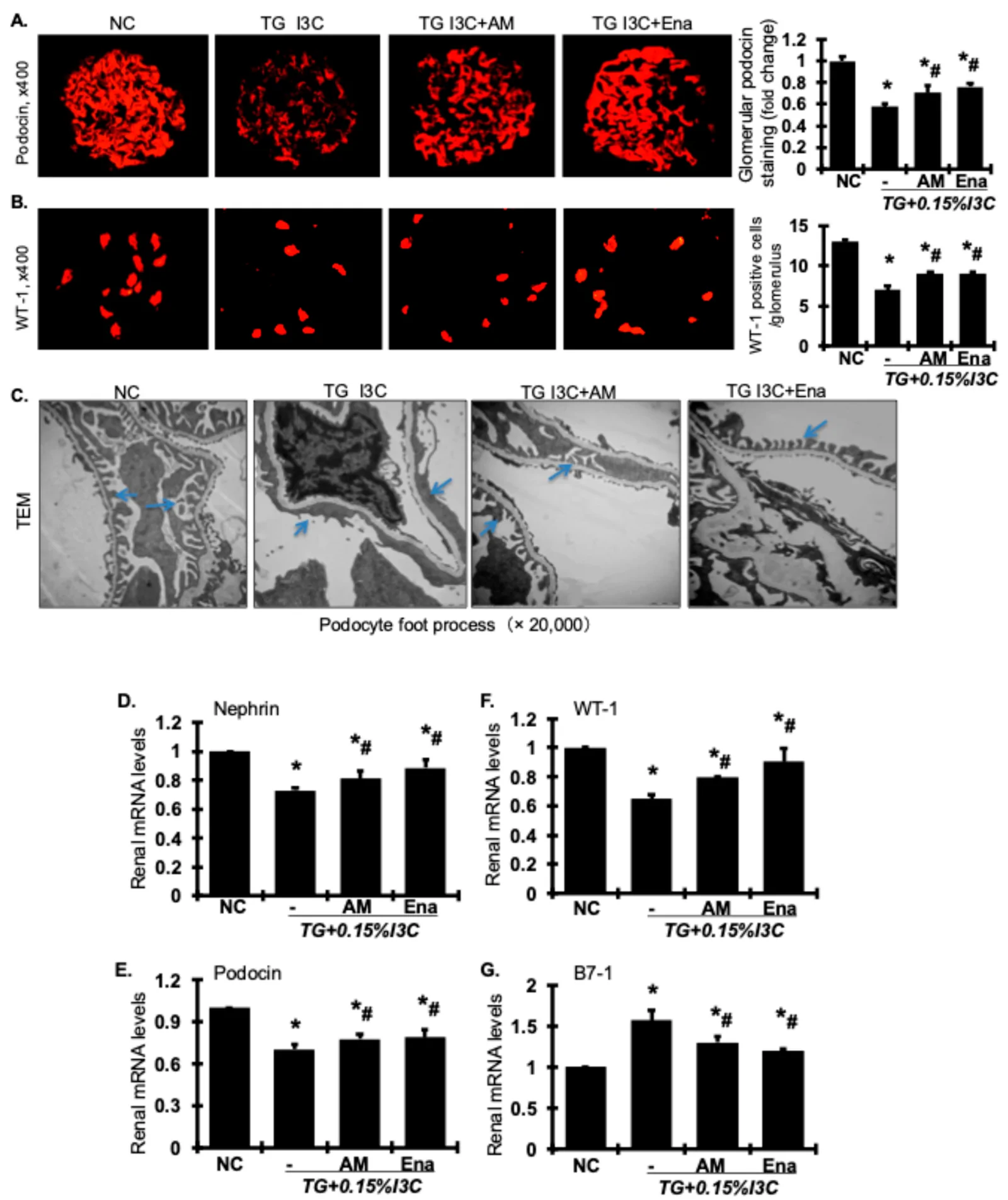
Effect of treatment with amlodipine and enalapril on prorenin-induced loss of podocyte markers and podocyte depletion in cyp1a1-prorenin transgenic rats. (**A**,**B**) Representative immunofluorescence staining of podocin ((**A**), top panels) and WT-1-positive podocyte nuclei ((**B**), middle panels) in glomeruli from normal control rats and prorenin-overexpressing rats (0.15%I3C-induced TG rats, TG I3C) treated without or with amlodipine (AM) or enalapril (Ena). Quantitative analyses of podocin fluorescence intensity and WT-1-positive cell counts are shown in the corresponding panels. *p* < 0.05 versus normal control (NC); * *p* < 0.05 versus untreated prorenin-overexpressing rats (TG + 0.15% I3C). (**C**) Representative transmission electron microscopy (TEM) images (lower panels) show that treatment with either AM or Ena ameliorates prorenin overexpression-induced segmental foot process effacement and widening of foot processes (as arrows pointed), compared with untreated transgenic animals. (**D**–**G**) Renal mRNA expression of nephrin, podocin, WT-1, and B7-1 was determined by real-time RT-PCR and normalized to β-actin expression. Data are presented as mean ± SD. * *p* < 0.05 vs. normal control (NC); # *p* < 0.05 vs. untreated prorenin-transgenic rats (TG + 0.15% I3C). n = 5–6 per group.

Treatment with AM or Ena significantly improved podocyte injury in prorenin-overexpressing rats. Specifically, both treatments restored glomerular podocin expression and increased the number of podocytes, as indicated by WT-1–positive cells per glomerular area (Figure 7A,B). In addition, transmission electron microscopy demonstrated that AM and Ena markedly ameliorated podocyte foot process effacement (Figure 7C).

Consistent with these histologic and structural findings, real-time RT-PCR revealed that both treatments upregulated nephrin, podocin, and WT-1 mRNA expression, while suppressing B7-1 expression in renal cortical tissue (Figure 7D–G), which may contribute to reduced albuminuria after treatment. However, enalapril produced greater improvements in prorenin-induced podocyte injury than AM, restoring these markers to levels closer to those observed in normal controls.

In contrast, compared with non-transgenic wild-type controls, untreated prorenin-overexpressing rats exhibited reduced podocin expression, decreased podocyte number, marked foot process effacement, and dysregulated mRNA expression of podocyte injury markers (Figure 7A–G).

### 2.8. Effect of Treatment on Glomerular Fibrosis

**Figure 8:**
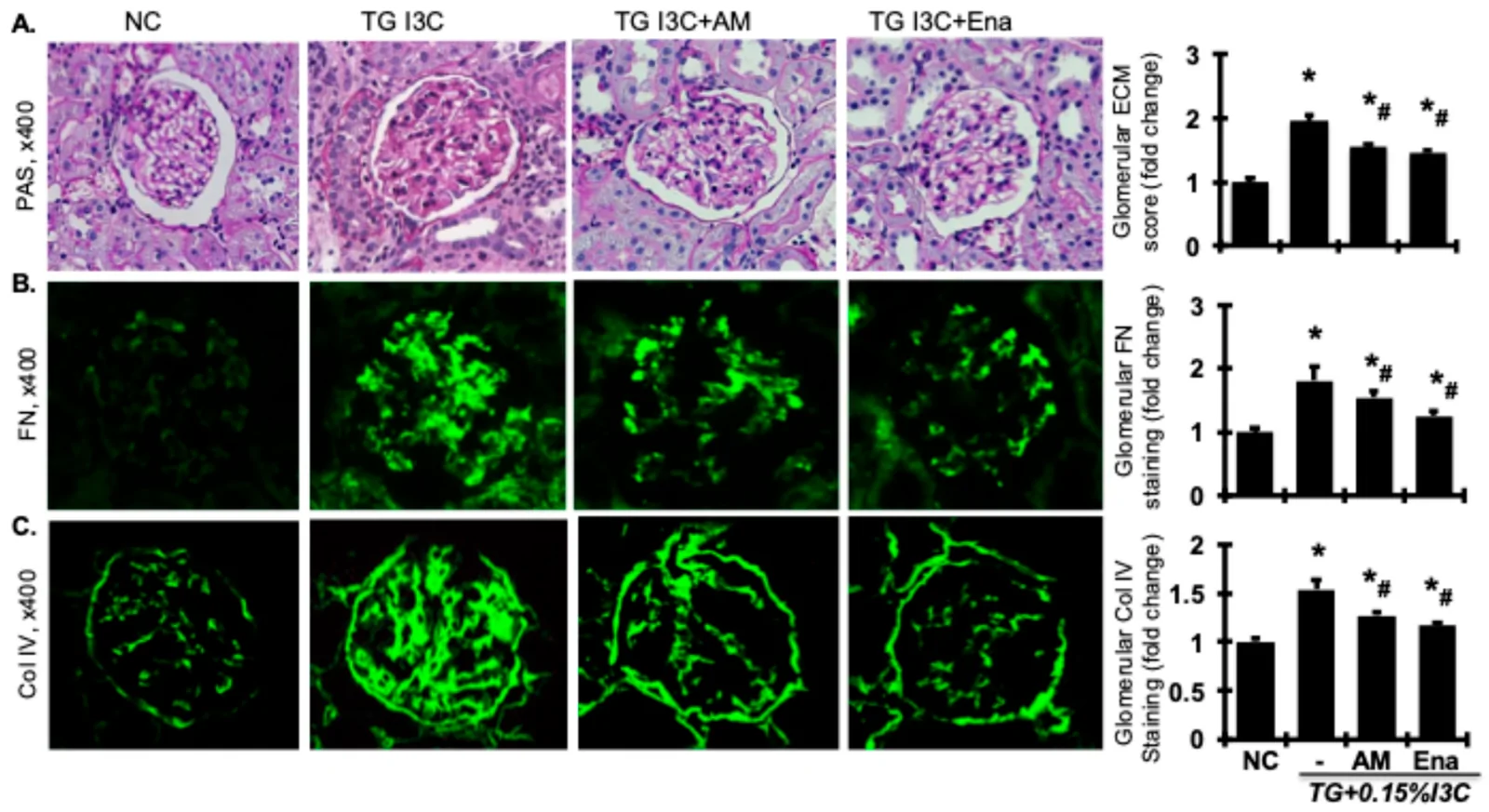
Effect of treatment with amlodipine and enalapril on glomerular fibrosis in cyp1a1-prorenin transgenic rats. Representative photomicrographs of periodic acid–Schiff (PAS) staining ((**A**), top panels), fibronectin (FN) immunofluorescence staining ((**B**), middle panels), and collagen IV (Col-IV) immunofluorescence staining ((**C**), bottom panels) in kidneys from normal control rats (NC) and prorenin-overexpressing rats (0.15%I3C-induced transgenic rats (TG)) treated with or without amlodipine (AM) or enalapril (Ena). Quantitative analyses of glomerular injury scores and fibrotic marker staining are presented in the corresponding panels. Data are presented as mean ± SD. * *p* < 0.05 vs. normal control (NC); # *p* < 0.05 vs. untreated prorenin-transgenic rats (TG + 0.15% I3C). n = 5–6 per group.

As expected, treatment with amlodipine (AM) or enalapril (Ena) significantly reduced glomerular extracellular matrix (ECM) accumulation, including FN and collagen IV (Col IV) deposition, compared with untreated prorenin-overexpressing rats (Figure 8A–C).

**Figure 9:**
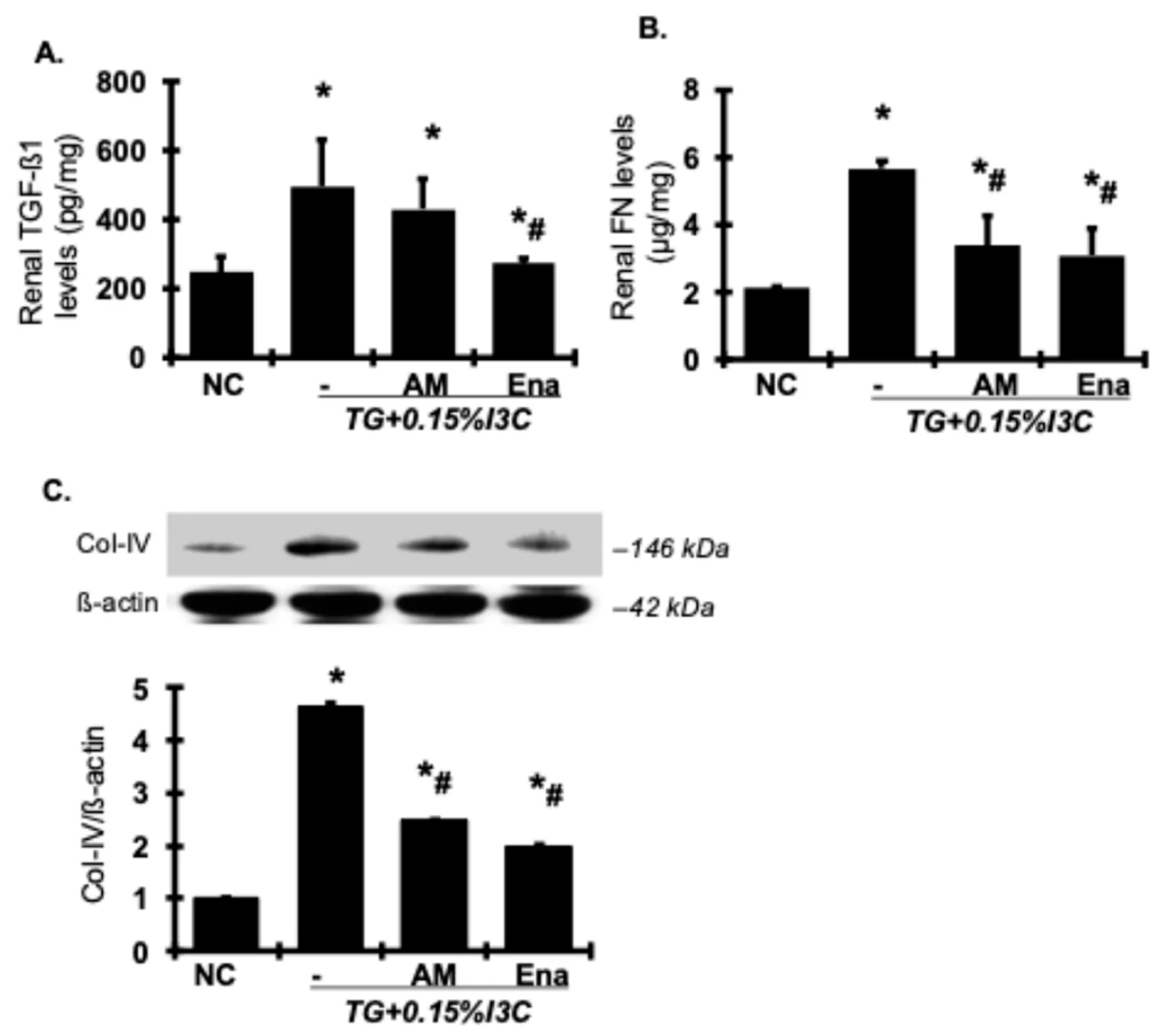

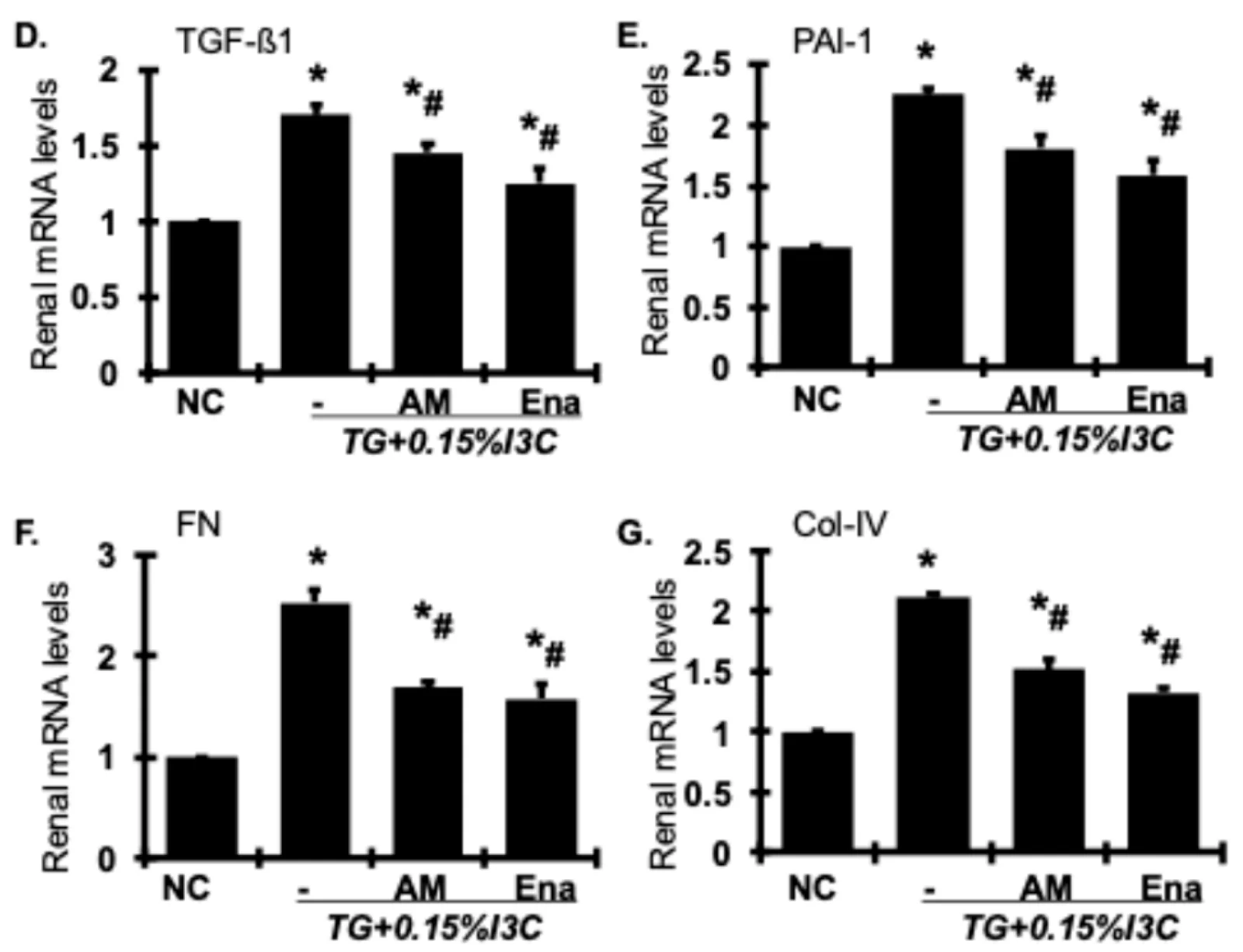
Effect of treatment with amlodipine and enalapril on prorenin-induced profibrotic marker expression in cyp1a1-prorenin transgenic rats. (**A**,**B**) Renal cortex levels of TGF-β1 and FN were determined by ELISA. (**C**) Representative Western blot analyses of Col-IV and β-actin protein expression in renal cortex tissues. β-actin was used as a loading control. Quantitative densitometric analyses of the corresponding Western blots are shown below the blot panels. (**D**–**G**) mRNA expression levels of TGF-β1, PAI-1, FN and Col-IV in renal cortex tissue were determined by real-time RT-PCR and normalized to β-actin expression. Data are presented as mean ± SD. * *p* < 0.05 vs. normal control (NC); # *p* < 0.05 vs. untreated prorenin-transgenic rats (TG+0.15%I3C). n = 5–6 per group.

Consistently, analysis of renal profibrotic markers by ELISA and Western blot demonstrated that AM and Ena significantly decreased prorenin-induced expression of TGF-β1, FN, and Col IV (Figure 9A–C). However, the levels of these markers remained elevated compared with normal controls. Except for FN, most markers showed greater reduction in Ena-treated prorenin transgenic rats than in AM-treated rats. Renal mRNA expression patterns were consistent with protein-level findings (Figure 9D–G).

Together, these results indicate that prorenin-induced renal fibrosis is attenuated, but not fully normalized, by treatment with AM or Ena.

### 2.9. Effect of Treatment on NF-kB and Nox2 Signaling Pathways

We have previously shown that nuclear factor kappa-light-chain-enhancer of activated B cells (NF-κB) and nicotinamide adenine dinucleotide phosphate (NADPH) oxidase 2 (Nox2)-mediated inflammatory and oxidative signaling pathways contribute to prorenin-induced renal and cardiac fibrosis [10]. Here, we further examined whether modulation of these pathways underlies the therapeutic effects of treatment.

**Figure 10:**
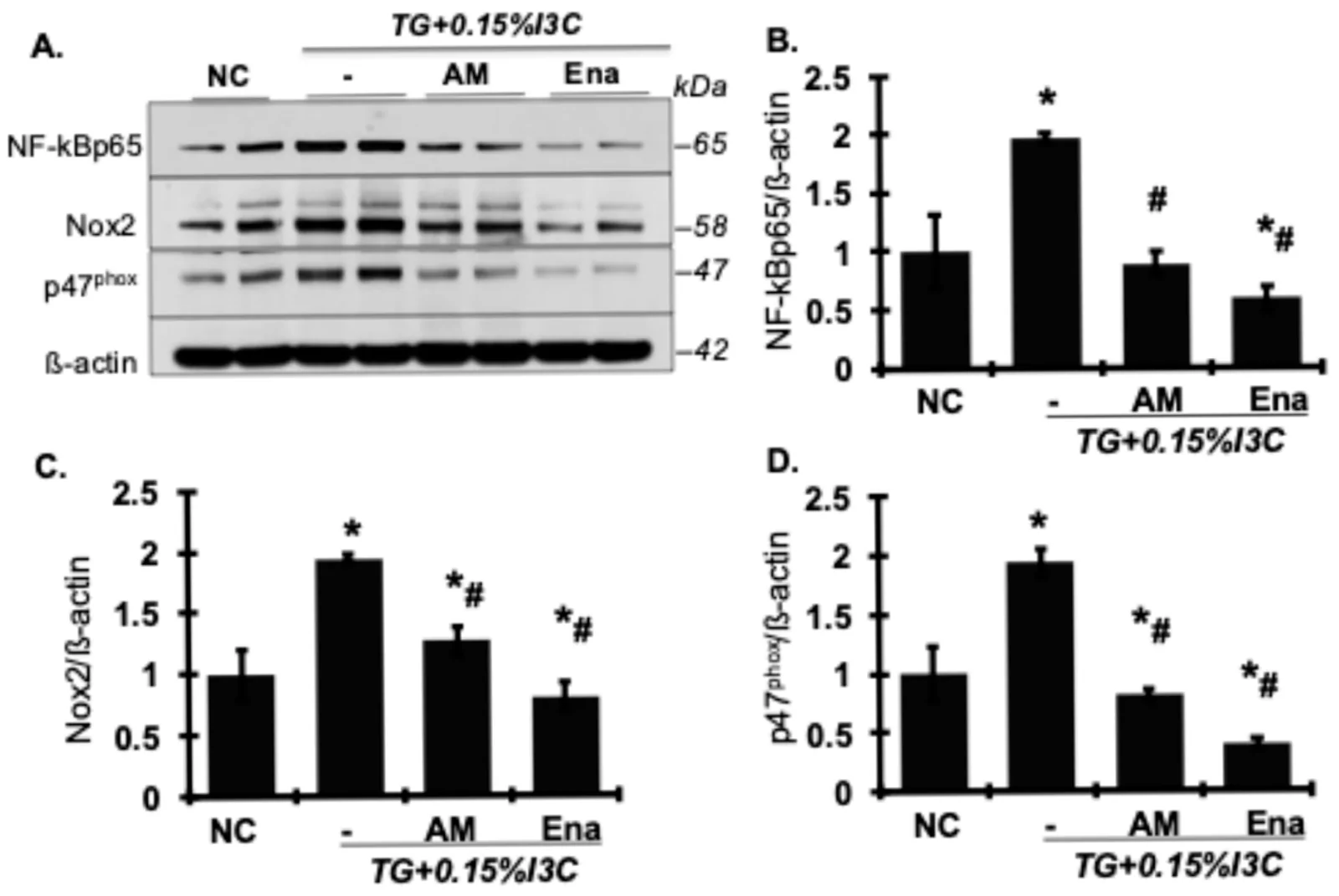
Effect of treatment with amlodipine and enalapril on inflammatory and oxidative stress signaling pathways in cyp1a1-prorenin transgenic rats. (**A**) Representative Western blot analyses of NF-kBp65, Nox2, p47phox, and β-actin protein expression in renal cortex tissues. β-actin was used as a loading control. (**B**–**D**) Quantitative densitometric analyses of the corresponding Western blots are shown in the corresponding panels. Data are presented as mean ± SD. * *p* < 0.05 vs. normal control (NC); # *p* < 0.05 vs. untreated prorenin-transgenic rats (TG + 0.15% I3C). n = 5–6 per group.

As shown in Figure 10, elevated prorenin markedly increased renal expression of NF-κBp65, Nox2, and its cofactor p47^phox^ in prorenin-overexpressing rats. Treatment with AM significantly attenuated these increases, whereas Ena produced an even greater inhibitory effect, reducing the expression of these markers to levels below those observed in wild-type control rats.

These findings suggest that the protective effects of AM and Ena on prorenin-induced podocyte injury and glomerular fibrosis are mediated, at least in part, through inhibition of NF-κB- and Nox2-dependent inflammatory and oxidative stress pathways.

## 3. Discussion

In the present study, we demonstrate that chronic elevation of circulating prorenin is sufficient to induce podocyte injury, albuminuria, and glomerular fibrosis in a dose-dependent manner in cyp1a1–prorenin transgenic rats. Using an inducible liver-specific model of prorenin overexpression, we show that increased plasma prorenin causes hypertension, loss of podocyte slit diaphragm proteins, podocyte depletion, and ultrastructural abnormalities, including podocyte foot process effacement and podocyte hypertrophy. Notably, structural injury, including podocyte loss and extracellular matrix accumulation, exhibited a clear dose-dependent relationship with circulating prorenin levels. In contrast, functional parameters such as albuminuria and renal function showed a less linear response, suggesting that structural injury may precede measurable functional decline. These findings provide direct evidence that elevated circulating prorenin is not merely a biomarker associated with kidney disease but is itself a pathogenic mediator of glomerular injury [6].

Elevated plasma prorenin levels have long been associated with diabetic microvascular complications, including diabetic nephropathy, and recent findings from the CRIC study further demonstrated an independent association between circulating prorenin, diabetes, and reduced kidney function [2,3,6]. However, whether elevated prorenin directly contributes to kidney injury or simply reflects underlying disease processes has remained unclear. By selectively increasing circulating prorenin in the absence of hyperglycemia, the present study establishes a causal link between elevated prorenin and progressive glomerular injury.

Importantly, clinical studies have reported that circulating prorenin levels are increased approximately two- to five-fold in patients with diabetes and diabetic kidney disease [22,23]. Elevated urinary prorenin has also been reported in patients with diabetic kidney disease, consistent with activation of the intrarenal prorenin/renin system [22]. In the present study, induction of the Cyp1a1-prorenin transgenic rats with 0.15% I3C produced an approximately 6-fold increase in circulating prorenin, closely matching the pathological range observed in patients with diabetic kidney disease. Thus, our model reproduces clinically relevant circulating prorenin concentrations and provides a physiologically relevant platform for investigating the direct pathogenic effects of elevated prorenin independent of hyperglycemia. Together, these findings support the concept that increased prorenin may actively contribute to CKD progression and suggest that prorenin represents a mechanistic link between diabetes and kidney injury.

A major finding of this study is the marked susceptibility of podocytes to elevated circulating prorenin. Podocyte injury is increasingly recognized as a central event in the initiation and progression of glomerular diseases, including diabetic nephropathy [24]. The observed reductions in nephrin and podocin expression, decreased WT-1–positive podocyte numbers, and ultrastructural evidence of foot process effacement indicate that prorenin disrupts podocyte integrity and filtration barrier function. Furthermore, the biphasic changes in creatinine clearance, characterized by an early increase at lower levels of prorenin induction, are suggestive of glomerular hyperfiltration, a hallmark of early diabetic kidney disease [25]. These findings suggest that podocytes represent a primary cellular target of excess circulating prorenin and may account for the development of albuminuria and subsequent glomerulosclerosis in diabetes.

The mechanisms underlying prorenin-mediated kidney injury likely involve both Ang II-dependent and Ang II-independent pathways. The ability of enalapril and amlodipine to prevent hypertension and partially attenuate renal injury indicates that hemodynamic stress and activation of the renin–angiotensin system contribute to disease progression. However, persistent albuminuria, podocyte injury, and fibrosis despite normalization of blood pressure suggest that elevated prorenin exerts additional pathogenic effects beyond systemic blood pressure regulation. Previous studies have demonstrated that binding of prorenin to the (pro)renin receptor (PRR) activates intracellular signaling pathways, including MAPK, PI3K, and TGF-β signaling, independent of Ang II generation [7,9,10,13]. Importantly, PRR is highly expressed in podocytes, where it is essential for maintaining cellular homeostasis and survival [11,12]. Although treatment with AM or Ena largely reversed prorenin overexpression-induced upregulation of PRR, these treatments did not reduce the marked elevation in circulating prorenin levels and therefore were unlikely to fully suppress activation of the prorenin–PRR signaling axis. Consistent with this interpretation, substantial podocyte injury and glomerular fibrosis persisted despite treatment, suggesting that direct prorenin–PRR signaling may contribute to renal injury independently of blood pressure elevation and Ang II generation. Thus, while PRR serves critical physiological functions, our findings are consistent with previous studies implicating the prorenin–PRR axis in kidney injury [13,14,15] and extend these observations by demonstrating that chronic elevation of circulating prorenin is sufficient to promote podocyte injury and glomerular fibrosis in vivo. Together, these findings support the concept that excessive activation of the prorenin–PRR axis by elevated circulating prorenin may trigger pathological signaling that promotes glomerular injury and disease progression.

Our data further implicate oxidative stress and inflammation as important downstream mediators of prorenin-induced kidney injury. Increased expression of Nox2 and p47phox suggests activation of NADPH oxidase-dependent reactive oxygen species production. Although we did not measure malondialdehyde (MDA), a marker of lipid peroxidation and oxidative stress, in the present study, we previously demonstrated that increased Nox2 expression in this prorenin transgenic rat model was accompanied by significantly elevated renal MDA levels, providing direct evidence of enhanced oxidative stress [10]. Furthermore, upregulation of NF-κBp65 indicates activation of proinflammatory transcriptional programs. These findings are consistent with previous reports linking prorenin–PRR activation to oxidative stress and inflammatory signaling [10,13,26,27,28]. However, treatment with either amlodipine or enalapril largely suppressed the induction of these pathways, with enalapril generally producing a greater inhibitory effect. Despite this attenuation, substantial podocyte injury and glomerular fibrosis persisted, suggesting that oxidative stress and inflammation alone do not fully account for the pathogenic effects of elevated prorenin. These findings raise the possibility that the prorenin–PRR axis promotes kidney injury through additional signaling pathways, including direct effects on podocyte survival, cytoskeletal integrity, and profibrotic responses, independent of its actions on oxidative stress and inflammation.

The inducible cyp1a1–prorenin transgenic rat model used in this study provides several unique advantages for investigating the pathogenic role of circulating prorenin. Unlike diabetic models, in which multiple metabolic and hemodynamic abnormalities coexist, this inducible system allows selective elevation of plasma prorenin independent of hyperglycemia. The observed dose-dependent relationship between circulating prorenin levels and glomerular injury further strengthens the causal link between prorenin and kidney damage. Nevertheless, the interaction between elevated prorenin and the metabolic abnormalities characteristic of diabetes warrants further investigation and may be particularly relevant to the progression of diabetic kidney disease.

The incomplete protection achieved with enalapril and amlodipine has important therapeutic implications. Although blockade of the renin–angiotensin system remains a cornerstone of CKD and diabetic nephropathy treatment, these interventions did not completely prevent podocyte injury or glomerular fibrosis in the present study. These findings suggest that targeting Ang II generation or blood pressure alone may be insufficient to fully suppress the pathogenic effects of elevated prorenin. Therapeutic strategies directed at prorenin itself or downstream PRR-mediated signaling pathways may therefore provide additional renoprotective benefits.

In conclusion, our findings demonstrate that chronic elevation of circulating prorenin directly promotes podocyte injury and glomerular fibrosis through both Ang II-dependent and Ang II-independent mechanisms. By establishing a causal relationship between elevated prorenin and progressive glomerular injury, this study provides new insight into the pathogenic role of the prorenin–PRR axis in CKD and diabetic kidney disease. These findings suggest circulating prorenin as an active mediator of kidney injury and support the development of therapeutic approaches targeting prorenin–PRR signaling to prevent progressive renal fibrosis and kidney failure.

## 4. Materials and Methods

Unless specified, all reagents were purchased from Sigma Chemical Co. (St. Louis, MI, USA).

Animals

All breeding, animal husbandry, and experimental procedures were conducted at the University of Utah in accordance with the Public Health Service Policy on Humane Care and Use of Laboratory Animals and the Guide for the Care and Use of Laboratory Animals. All protocols were approved by the Institutional Animal Care and Use Committee (IACUC) of the University of Utah before initiation of the study.

Generation of cyp1a1-prorenin transgenic rats and induction of transgene expression by I3C.

The inducible cyp1a1–prorenin transgenic rat line, in which the cytochrome P450 1A1 (cyp1a1) promoter drives liver-specific transgene expression, has been established and maintained in our laboratory as previously described [10]. cyp1a1 is not constitutively expressed but is robustly induced upon activation of aryl hydrocarbon receptor-dependent transcriptional pathways, whereby ligand-activated transcription factors bind to response elements within the cyp1a1 promoter [17,18].

Indole-3-carbinol (I3C), a naturally occurring compound found in cruciferous vegetables, serves as a well-tolerated inducer with a short biological half-life [16]. Induction of cyp1a1 promoter–driven transgene expression by dietary I3C has been previously validated in this model [10]. For experimental studies, I3C was incorporated into standard rat chow at varying concentrations (Harlan Laboratories, Madison, WI, USA) and administered to transgenic rats to achieve graded levels of prorenin expression.

Experimental design

*Study 1 (dose–response of prorenin).*

To determine the dose-dependent effects of prorenin on glomerular podocyte injury, cyp1a1–prorenin transgenic male rats (12 weeks of age) were randomized into three groups and fed diets containing incremental concentrations of the transgene inducer indole-3-carbinol (I3C): 0.05% (*w*/*w*; n = 6), 0.15% (n = 6), or 0.3% (n = 4) for 4 weeks. Age-matched wild-type rats fed standard chow (n = 6) served as healthy controls.

*Study 2 (Ang II-dependent mechanisms).*

To determine the Ang II-dependent mechanisms of prorenin, we used enalapril (an ACE inhibitor that blocks Ang II generation). Based on results from Study 1, the optimal I3C dose (0.15%) for inducing plasma prorenin, podocyte injury, and glomerular fibrotic markers was selected. At 12 weeks of age, transgenic male rats were fed chow containing 0.15% I3C and randomized into three groups: untreated (n = 5, disease control), enalapril-treated (200 mg/L in drinking water, changed daily; n = 6), or amlodipine-treated (10 mg/kg/day by oral gavage; n = 6, blood pressure control). The doses of enalapril and amlodipine were selected based on their previously established maximal efficacy for inhibition of Ang II generation and blood pressure control [20]. Treatment was initiated simultaneously with prorenin induction and continued for 6 weeks. Wild-type rats fed standard chow (n = 5) served as normal controls. All animals had free access to food and water.

No animals or experimental units were excluded from Study 1 and Study 2. No inclusion or exclusion criteria were established a priori. All animals completed the experimental protocol and were included in the final analyses. The exact sample size (n) for each group is reported in the corresponding figure legends and tables.

Previous studies from our laboratory demonstrated that feeding wild-type rats (both males and females) a diet containing 0.3% indole-3-carbinol (I3C) for 4 weeks did not alter body weight, blood pressure, renal function, renal histological structure, or renal inflammatory changes compared with wild-type rats maintained on standard chow [10]. Similarly, transgenic rats without I3C induction exhibited physiological and renal parameters comparable to those of wild-type controls, indicating that a 0.3% I3C diet alone does not produce significant physiological or renal effects under these experimental conditions. Based on these previously validated findings, wild-type rats maintained on a standard diet were used as the control group in both Study 1 and Study 2.

Body weight (BW) was recorded at baseline prior to prorenin induction and intensive treatment and at the end of the study. Blood pressure was measured by tail-cuff plethysmography as previously described [10]. At study completion, 24 h urine samples were collected using metabolic cages. Urinary albumin and albumin-to-creatinine (A/C) ratio were measured using the DC2000+ microalbumin assay kit (Wilburn Medical Inc., Kernersville, NC, USA). Twenty-four-hour urinary albumin excretion was calculated by multiplying the urinary albumin concentration by the total 24 h urine volume and is expressed as mg/day.

At the end of each experiment, rats were anesthetized with isoflurane and euthanized. Mean arterial pressure (MAP) was measured via catheterization of the lower abdominal aorta using a Digi-Med Blood Pressure Analyzer (BPA-400, Micro-Med, Inc., Louisville, KY, USA). Blood (5–10 mL) was collected, and organs were perfused with 60 mL ice-cold PBS. Kidneys and hearts were excised, weighed, and the kidney weight-to-body weight (KW/BW) ratio and the heart weight-to-body weight (HW/BW) ratio were calculated.

Renal cortex tissue was dissected and processed for downstream analyses as previously described [10]. Briefly, cortical samples were (i) snap-frozen in 2-methylbutane and stored at −80 °C; (ii) fixed in 10% neutral buffered formalin for histological and immunohistochemical analyses; or (iii) fixed in 2.5% glutaraldehyde in 0.1 M sodium cacodylate buffer for electron microscopy. Additional renal cortex and liver tissues were snap-frozen in liquid nitrogen for protein and RNA analyses. For protein ELISA assay, tissues were homogenized in buffer (100 mM NaCl, 20 mM HEPES), sonicated on ice (3 × 30 s), and centrifuged at 12,000 rpm for 15 min at 4 °C. Supernatants were collected and stored at −80 °C for fibronectin (FN) and TGF-β1 ELISA (R&D Systems, Minneapolis, MN, USA) [21,29], and protein concentration was determined using a bicinchoninic acid (BCA) assay (Pierce).

Determination of renal function

Plasma or urine BUN and creatinine (Cr) concentrations were measured by using the QuantiChromTM urea assay kit and creatinine assay kit (BioAssay System, Hayward, CA, USA). The creatinine clearance (Ccr) was calculated as the following formula: (urine creatinine levels/plasma creatinine levels) × 24 h-urine volume (mL)/(24 h × 60 min).

Histological analysis

Formalin-fixed renal cortex tissues were embedded in paraffin, and 3 µm sections were cut and stained with periodic acid–Schiff (PAS). PAS-positive glomerular extracellular matrix was quantified in a blinded manner using a computer-assisted image analysis system as previously described [30]. At least 20 glomeruli per rat were analyzed at high magnification (×400). Mesangial matrix area (PAS-positive, magenta staining) was quantified as optical density (OD) using ImageJ (NIH-ImageJ 1.53t, Bethesda, MD, USA), and values from diseased or treated groups were normalized to the control group, which was set to one.

Immunofluorescence staining for nephrin, podocin, Wilms tumor protein 1 (WT-1), fibronectin (FN), collagen type I (Col I), and collagen type IV (Col IV) was performed on frozen sections. Primary antibodies included goat anti-nephrin (catalog No. sc-32530, Santa Cruz Biotechnology Inc., Dallas, TX, USA), goat anti-podocin (catalog No. sc-22298, Santa Cruz Biotechnology Inc.), and rabbit anti-WT-1 (C-19) (catalog No. sc-192, Santa Cruz Biotechnology Inc.), rabbit anti-fibronectin (catalog No, F3648, Sigma-Aldrich, Inc., St. Louis, MO, USA), goat anti–collagen I (catalog No. 1310-01, SouthernBiotech, Birmingham, AL, USA), and rabbit anti-collagen IV (catalog No. 600-401-106, Rockland Immunochemicals Inc., Pottstown, PA, USA). Appropriate secondary antibodies conjugated to TRITC, Alexa Fluor 594, or FITC (Jackson ImmunoResearch, West Grove, PA, USA or Invitrogen, Carlsbad, CA, USA) were used.

Glomerular fluorescence intensity was quantified in a blinded manner using a computer-assisted method as previously described [31]. Data from diseased or treated groups were normalized to the control group (set as one). The number of WT-1-positive podocytes per glomerulus was determined by counting at least 20 randomly selected glomeruli per section.

Transmission electron microscopy

Glutaraldehyde-fixed renal cortex tissues were post-fixed in osmium tetroxide (OsO_4_) and embedded in Epon 812. Ultrathin sections were cut, stained with uranyl acetate and lead citrate, and examined using a transmission electron microscope (JEOL JEM-1010, Tokyo, Japan). Podocyte ultrastructure, including foot process morphology, was evaluated.

Western blot analysis

Renal cortex tissue (15 mg) from each rat was homogenized in lysis buffer (Cell Signaling Technology, Beverly, MA, USA) containing 1% NP-40, 1 mM PMSF, and protease inhibitor cocktail (Complete Mini, Roche Diagnostics, Indianapolis, IN, USA). Equal amounts of protein from each rat within a group were pooled to generate a representative sample for that group.

For Western blot analysis, protein samples (20 µg) were separated by SDS-PAGE using 4–12% gradient gels (Invitrogen) and transferred to Immobilon-P membranes (Millipore, Burlington, MA, USA). Membranes were probed with antibodies against prorenin/renin, podocin (catalog No. sc-22298, Santa Cruz Biotechnology, Inc.), nephrin (catalog No. TA306037, OriGene Technologies, Inc., Rockville, MD, USA), B7-1 (catalog No. TA303430, OriGene Technologies, Inc.), plasminogen activator inhibitor-1 (PAI-1) (catalog No. 612025, BD Biosciences, San Jose, CA, USA), collagen type I (Col I) (catalog No. 1310-01, SouthernBiotech), collagen type IV (Col IV) (catalog No. 600-401-106, Rockland Immunochemicals Inc.) nuclear factor-κB-p65 (NF-κBp65) (catalog No. 6956, Cell Signaling Technology, Beverly, MA, USA), NADPH oxidase gp91^phox^ (Nox2) (catalog No. 611414, BD Biosciences), p47^phox^ (catalog No. 610354, BD Biosciences), and β-actin (catalog No. A5441, Sigma-Aldrich). Immunoreactive bands were detected and quantified as previously described [10,31,32,33].

Circulating prorenin levels were assessed by loading 2 µL of plasma from each rat diluted in 18 µL lysis buffer (Cell Signaling Technology), followed by SDS-PAGE and immunoblotting as described above [10]. Equal sample loading was ensured by loading identical plasma volumes for all samples and was verified by Ponceau S staining of the membrane after protein transfer. Representative Western blot images were derived from pooled samples for each group.

RNA preparation and quantitative real-time RT-PCR

Total RNA was isolated from renal cortex and heart tissues using TRI Reagent according to the manufacturer’s instructions. RNA concentration and purity were assessed spectrophotometrically. Two micrograms of total RNA were reverse-transcribed using the Superscript III First-Strand Synthesis System (Invitrogen).

Quantitative real-time RT-PCR (qRT-PCR) was performed using SYBR Green I chemistry (Applied Biosystems, Waltham, MA, USA) on an ABI 7900 Sequence Detection System, as previously described [34]. Samples were run in triplicate, and gene expression levels were normalized to β-actin as an internal control using the comparative Ct (ΔΔCt) method. Primer sequences for podocyte injury markers (nephrin, podocin, WT-1, B7-1), fibrotic markers (TGF-β1, PAI-1, fibronectin, collagen I, collagen IV), (pro)renin receptor (PRR), and β-actin have been reported previously [10,31,35]. PCR specificity was confirmed by agarose gel electrophoresis (1.5%) showing a single band of the expected size.

Statistical analysis

Data are presented as mean ± SD, with n representing the number of animals. Statistical comparisons among multiple groups were performed using one-way ANOVA followed by Student–Newman–Keuls or Dunnett’s post hoc tests, as appropriate. A *p*-value < 0.05 was considered statistically significant.

## Figures and Tables

**Table 1:** Experimental parameters of the rat dose groups.

Parameters	WT NC (n = 6)	TG+ 0.05%I3C (n = 6)	TG+ 0.15%I3C (n = 6)	TG+ 0.3%I3C (n = 4)
Δ body wt, g	89.06 ± 6.32	10.07 ± 8.95 *	24.87 ± 14.32 * #	−58.43 ± −28.18 * # §
Systolic Bp (SBP), initial, mmHg	84.83 ± 7.28	86.36 ± 6.50	85.62 ± 5.69	87.87 ± 10.94
MAP, final, mmHg	82.98 ± 10.94	130.50 ± 60.72 *	126.56 ± 27.49 *	140.87 ± 30.74 *
Plasma BUN, mg/dL	16.46 ± 1.20	27.66 ± 7.83 *	28.24 ± 3.66 *	28.94 ± 3.56 *
Plasma creatinine, mg/dL	0.35 ± 0.05	0.43 ± 0.10	0.44 ± 0.17	0.65 ± 0.34 *
Ccr, mL/min	2.10 ± 0.59	2.945 ± 0.71	1.82 ± 0.64	1.73 ± 1.39
urinary albumin excretion (UAE), final, mg/d	0.168 ± 0.07	22.39 ± 12.30 *	21.06 ± 10.00 *	29.24 ± 10.63 *
urine albumin/creatinine (A/C), final, mg/g	23.11 ± 9.04	974.50 ± 366.37 *	857.00 ± 231.65 *	1064.66 ± 375.55 *
kidney wt/body wt, final, mg/g	3.42 ± 0.35	4.30 ± 1.1	5.94 ± 0.99 * #	6.17 ± 0.77 * #
Heart wt/body wt, final, mg/g	2.97 ± 0.26	5.08 ± 1.40 *	5.52 ± 0.88 *	6.33 ± 1.35 * # §

Δ, 0. vs. normal controls; * *p* < 0.05 vs. normal controls; # *p* < 0.05 vs. 0.05% I3C-induced transgenic (TG) rats; § *p* < 0.05 vs. 0.15% I3C-induced TG rats. n = 4–6 per group.

**Table 2:** Experimental parameters of transgenic rat groups with or without treatment.

Parameters	WT-NC (n = 5)	TG + I3C (n = 5)	TG + I3C +AM (n = 6)	TG + I3C +Ena (n = 6)
Δ body wt, g	134.44 ± 16.92	21.25 ± 12.33 *	87.60 ± 48.57 * #	101.44 ± −48.88 #
Systolic Bp (SBP), initial, mmHg	84.83 ± 7.28	86.36 ± 6.50	85.62 ± 5.69	87.87 ± 10.94
MAP, final, mmHg	91.7 ± 7.29	132.4 ± 4.77 *	63.41 ± 5.78 * #	85.96 ± 16.39 #
UAE, final, mg/d	0.23 ± 0.07	9.35 ± 3.35 *	1.11 ± 0.33 * #	0.31 ± 0.14 #
Urine A/C, final, mg/g	17.5 ± 5.52	535.86 ± 347.45 *	91.8 ± 27.60 * #	25.36 ± 38.91 #
Plasma BUN, mg/dL	16.34 ± 1.32	31.20 ± 3.21 *	27.09 ± 7.92 *	23.95 ± 3.90 * #
Ccr, mL/min	1.98 ± 0.95	1.81 ± 0.41	2.13 ± 0.43	2.83 ± 1.23 #
kidney wt/body wt, final, mg/g	3.19 ± 0.23	4.88 ± 0.51 *	4.29 ± 0.91 * #	4.28 ± 0.34 * #
Heart wt/body wt, final, mg/g	2.65 ± 0.19	4.94 ± 0.19 *	4.19 ± 0.63 * #	3.37 ± 0.32 * #

Δ, 0. vs. normal control (NC); * *p* < 0.05 vs. normal controls; # *p* < 0.05 versus untreated prorenin-transgenic rats (TG + 0.15% I3C).

## Data Availability

Please contact the corresponding author for data requests.
